# Leaf bacterial microbiota response to flooding is controlled by plant phenology in wheat (*Triticum aestivum* L.)

**DOI:** 10.1038/s41598-022-15133-6

**Published:** 2022-07-01

**Authors:** Davide Francioli, Geeisy Cid, Mohammad-Reza Hajirezaei, Steffen Kolb

**Affiliations:** 1 Microbial Biogeochemistry, Research Area Landscape Functioning, Leibniz Center for Agricultural Landscape Research E.V. (ZALF), Müncheberg, Germany; 2 Department of Physiology and Cell Biology, Leibniz Institute of Plant Genetics and Crop Plant Research, Gatersleben, Germany; 3 Thaer Institute, Faculty of Life Sciences, Humboldt University of Berlin, Berlin, Germany

**Keywords:** Agroecology, Microbial ecology

## Abstract

Leaf microbiota mediates foliar functional traits, influences plant fitness, and contributes to various ecosystem functions, including nutrient and water cycling. Plant phenology and harsh environmental conditions have been described as the main determinants of leaf microbiota assembly. How climate change may modulate the leaf microbiota is unresolved and thus, we have a limited understanding on how environmental stresses associated with climate change driven weather events affect composition and functions of the microbes inhabiting the phyllosphere. Thus, we conducted a pot experiment to determine the effects of flooding stress on the wheat leaf microbiota. Since plant phenology might be an important factor in the response to hydrological stress, flooding was induced at different plant growth stages (tillering, booting and flowering). Using a metabarcoding approach, we monitored the response of leaf bacteria to flooding, while key soil and plant traits were measured to correlate physiological plant and edaphic factor changes with shifts in the bacterial leaf microbiota assembly. In our study, plant growth stage represented the main driver in leaf microbiota composition, as early and late plants showed distinct bacterial communities. Overall, flooding had a differential effect on leaf microbiota dynamics depending at which developmental stage it was induced, as a more pronounced disruption in community assembly was observed in younger plants.

## Introduction

Plant–microbe interactions involve a great variety of microorganisms from multiple domains^1,2^, and such interactions are major drivers of ecosystem functions^3,4^. Plants provide a multitude of niches for the growth and proliferation of various microorganisms, including bacteria, fungi, protists, nematodes and viruses. These microorganisms form complex co-associations with plants, constituting the plant microbiota. The members of a plant microbiota have crucial roles in plant productivity and health, comprising beneficial, neutral and pathogenic microorganisms^5^.

The plant microbiota is generally defined by host species and genotype, compartment, and tissue location^6–8^, as well as by environmental factors, such as edaphic properties and climatic variables^9–12^. Leaf-colonizing microorganisms have been described as an extension of host phenotypes as they can impact plant fitness in several ways^13,14^. Pathogens that invade and infect leaves are examples of the adverse effects that microorganisms can have on plant fitness. On the contrary, commensal leaf microbes can positively influence plant fitness via diverse mechanisms, including growth promotion by hormones, modulation of the plant immune system, degradation of environmental pollutants, and enhancement of biotic and abiotic stress tolerance^15,16^. Indeed, foliar fungal endophytes can modulate resistance to pathogens^17,18^, and alter growth and survival rates of plant hosts exposed to drought, heat, and salinity^19–21^.

The composition and structure of leaf microbiota are key to its effects on the host, underlining the need to understand the mechanisms that govern the assembly and spatiotemporal dynamics of these communities^13,22^. Several recent studies have demonstrated that phyllosphere microbiota are not characterized by random microbial assemblies, but instead undergo selection that results, at least partially, in predictable bacterial communities dominated by few phyla^23,24^. Plant host species, environment and spatial factors were identified as key drivers in leaf bacteria community composition^25,26^. In *Arabidopsis thaliana*, soil type was found as the main driver of leaf bacterial community structure^27^, while several studies revealed that the association of leaf epiphytic and endophytic microbes differs greatly between plant hosts^28,29^. Furthermore, phyllosphere microbiota are highly variable over the growing season, although patterns in community composition were still detected^30–32^. These studies revealed that the leaf microbiota was characterized by season-specific bacterial taxa, thus inferring on particular mechanisms of leaf adaptation at different plant growth stages^33,34^. Collectively, these findings support the central role of plant phenology in leaf microbiota assembly, and the likelihood of functional and evolutionary changes associated with these predictable patterns^35,36^.

Climate change driven factors, such as weather extremes, were also found to be important determinants in leaf microbiota assembly, and it has been reported a high sensitivity of the phyllosphere to climate variables, such as temperature changes, drought and abnormal rain events^37,38^. Considering that seasonal trends are expected to shift towards a higher frequency of these extreme weather events with climate change^39,40^, this may have significant impacts on crop leaf microbiota assembly. As global climate change accelerates, there is an urge to improve our understanding of how plant–microbe interactions maintain terrestrial ecosystem productivity in the face of prolonged extreme weather events such as flooding and waterlogging.

Here, we performed a glasshouse experiment to investigate the response of the wheat bacterial leaf microbiota to flooding. Considering that (a) plant phenology is a major driver in shaping crop microbiota^7,41,42^ and (b) the microbial response to abiotic stress depends largely on the plant developmental stage in which the stress occurs^43,44^, wheat plants were subjected to flooding either at tillering, booting and flowering. To monitor the effect of flooding on the leaf bacteria community assembly, we employed metabarcoding targeting the 16S rRNA gene. We measured numerous soil and plant parameters to correlate physiological plant and edaphic changes with shifts in leaf bacterial community assembly. We hypothesized that (i) leaf microbiota composition and structure will be highly dynamic over plant growth stage (PGS) with marked differences between early and late bacterial communities and (ii) the leaf microbiota structure will be differentially affected by the timing of flooding events, with early assembly being more pronouncedly affected.

## Materials and methods

### Experimental setup

We performed a greenhouse experiment from September to December 2019 at the Leibniz Institute of Plant Genetics and Crop Plant Research (IPK) in Gatersleben (Germany) to investigate the response of the wheat microbiota to flooding stress. Seeds of spring wheat (*Triticum aestivum* L. Chinese Spring) were germinated under controlled conditions in 2 mm sieved soil, which was obtained from the experimental station in Dedelow (Germany). The soil is classified as a loamy sandy/medium silty sandy soil (S3/Su3 according to the German texture classification) Ad-hoc-AG-Boden^45^. Seedlings were individually transferred to 10L pots containing 5 kg of the soil used for germination (one seedling per pot) around the third week after sowing. Wheat plants were grown under controlled conditions of day/night temperature, i.e. 18/16 °C, light intensity 250–300 µmol/(m^2^ s), photoperiod of 16 h light/8 h darkness and air humidity 70%. A completely randomized design was employed to place the pots on glasshouse tables. To monitor the plant developmental stage, we utilized the Zadoks scale^46^. Flooding stress was induced only once and for 12 days, at either tillering (Z25–Z30), booting (Z37–Z49) or flowering (Z49–Z69) and replicates were destructively sampled (Fig. S1). Previous studies have shown that complete oxygen depletion in the top soil occurs within 2–8 days of flooding across a wide range of soils^47–49^. Thus, considering that the study aim was to explore the response of the leaf bacterial community to a severe and intense water stress, flooding was induced for a period of 12 days in order to ensure oxygen depletion in the flooded treatment. Six replicates were established for each combination of water treatment and plant growth stage (PGS), for a total of 36 pots. Control pots were maintained at 50% water holding capacity (the field capacity of the soil used), while flooding was induced by manually keeping water approximately 5 cm above the soil surface for 12 days.

### Plant and soil sampling

Control and flooded plants were harvested, and tillers and spikes number recorded, in the twelfth day of exposition to flooding in the corresponding developmental stages. Shoots and roots were separated, and both fresh and dry weight were measured. Samples from fully expanded leaves for molecular analysis were immediately frozen and stored at − 80 °C. Macro- (C, N, P, Mg, S, K and Ca) and micro-nutrients (Mn, Zn and Na) concentrations in the dry roots and leaves were measured using a sector field high-resolution mass spectrometer (HR)-ICP-MS (Element 2, Thermo Fisher Scientific, Germany). Several edaphic parameters were measured in the soil samples. Total organic carbon (TOC) and total nitrogen (TN) contents were measured in triplicate by dry combustion using a Vario EL III C/H/N analyser (Elementar, Hanau, Germany). Plant available P (PDL) was extracted from fresh soil using the double lactate extraction method (1:50 w/v, pH 3.6, 1.5 h; Riehm^50^), and the extracted P was quantified colorimetrically using the molybdenum blue method^51^. Soil Mn, Ca, Na, K, and Mg concentrations were determined with an inductively coupled plasma-optical emission spectrometry-ICP-OES (ICP-iCAP 6300 DUO, ThermoFisher Scientific, Germany).

### DNA extraction, library preparation and sequencing

DNA was extracted from the collected leaf samples using the DNeasy PowerLyzer PowerSoil Kit (Qiagen). Amplification of the bacterial DNA was performed following the PCR procedure as described previously in Francioli et al.^52^ using the primers 799f^53^ and 1115r^54^. In brief, PCR was carried out in a 50 μl reaction volume with 1 μl of DNA template (diluted 1:20 from the original extract), 0.2 mM dNTPs and 0.4 μM of each primer (PCR conditions: 95 °C for 5 min; 35 cycles at 95 °C for 1 min, 56 °C for 1 min and 72 °C for 1 min; and 72 °C for 5 min). The amplicons were sent to LGC Genomics GmbH (Berlin, Germany) for barcoding and paired-end sequencing on Illumina MiSeq v3 platform. Demultiplexing was performed using Illumina bcl2fastq v2.20 software (https://support.illumina.com/sequencing/sequencing_software/bcl2fastq-conversion-software.html) following clipping of barcode and sequencing adapters. Primer sequences were removed using Cutadapt v3.4^55^ following sequence processing using QIIME 2 v2021.2. DADA2 pipeline was employed to determine amplicon sequence variants (ASV) from the raw sequences^56^, with forward and reversed reads truncated at 200 bp and 150 bp, respectively. For the data analysis, we considered only ASVs that were detected in at least two samples. The representative sequence of each ASV were referenced against the naïve Bayesian classifier for SILVA 138 (https://www.arb-silva.de/documentation/release-138/), and non-bacterial ASVs were removed. Bacterial reads were rarefied to 20,000 reads per sample to calculate alpha diversity metrics.

### Statistical analyses

The soil and root data have been already published in a previous study^57^. They were used here together with the new, i.e. as yet unpublished, leaf traits data to create a new dataset of variables. We analysed this new dataset by multivariate analysis of variance to examine the contribution of all the measured variables on the leaf bacterial microbiota structure across PGS and between treatments.

Differences in plant traits and soil properties were tested among the treatments and PGS by univariate analysis of variance (ANOVA) followed by Tukey’s honestly significant difference (HSD) post hoc test. Normality and homogeneity of variance of the variables measured were examined with the Shapiro–Wilk and Levene’s test, respectively. All the variables that did not meet the parametric assumptions were log10 transformed. Spearman’s rank correlation was used to determine the correlation between the measured variables. Variables with a Spearman rank correlation coefficient ρ > 0.8 were excluded from further analysis. Effects of PGS and treatment on the leaf Bacteria richness were tested by generating univariate PERMANOVA models^58^. ANOVA followed by Tukey’s HSD post hoc test was further employed to perform pairwise comparisons on bacterial ASV richness between water treatments at the same PGS. Differences in Bacteria beta-diversity were determined across PGS and treatment. Community structure and bacterial community dissimilarity were calculated using Bray–Curtis distance based on the relative abundance data with Hellinger transformation (function *vegdist* in the R package “vegan”). To evaluate the effects of the experimental factors on the bacterial microbiota structure, we employed permutational multivariate analysis of variances (PERMANOVA) based on the Bray–Curtis dissimilarity using 999 permutations for each test. Variance partitioning based on redundancy analysis (RDA) was performed to quantify the contribution of plant and soil attributes, PGS and treatment to the structure of the leaf bacterial microbiota. The significance of the global model using all predictors was tested first, then we conducted variable selection using forward selection through the function *forward.sel* in the R package “packfor”^59^. Variance partitioning was performed with the *varpart* function in the “vegan” R package^60^. To determine the most important environmental variables correlated with leaf bacterial microbiota structure, we constructed a model of multivariate analysis of variance using distance-based redundancy analysis (db-RDA) based on the Bray–Curtis distance. Bacterial biomarker taxa characterizing flooding and control treatments at each PGS were identified by linear discriminant analysis effect size (LEfSe)^61^. Ternary plots were produced utilizing the package “microbiome utilities”^62^. All data were analyzed with R version 4.0^63^.

### Approval for plant experiments

*Triticum aestivum* L. Chinese Spring plants were used in this study, and their seeds were kindly provided by Prof. Andreas Börner (Leibniz Institute of Plant Genetics and Crop Plant Research, Gatersleben, Germany). No approvals were required for the cultivation of *Triticum aestivum* L. Chinese Spring, which complied with all relevant regulations.

## Results and discussion

We investigated how phyllosphere bacterial microbiota of the crop *Triticum aestivum* cv. Chinese Spring (i) developed over plant growth period and (ii) responded to flooding when such stress was induced at different plant growth stages (PGS). As plant phenology has been described as a major driver in wheat microbiota assembly^64,65^ and abiotic stress might have a differential impact on community assembly dynamics depending on the specific PGS^57^, we conducted a glasshouse experiment, in which wheat plants were subjected to a period of 12 days of flooding either at tillering, booting and flowering (Fig. S1). In the twelfth day of exposition to flooding, control and flooded plants of the corresponding PGS were removed from the experiment and harvested to collect a variety of soil, plant and microbiota associated traits to correlate changes in environmental and host traits with shifts in leaf microbiota alpha- and beta-diversity. To our knowledge, only a small number of studies have explored the dynamics of the leaf wheat microbiota over plant development^66–68^, whereas no study has yet investigated the response of wheat’s leaf bacterial microbiota to flooding stress. Since leaf microbiota influence plant fitness^69^, it is fundamental to resolve the host-microbe interactions in the phyllosphere of crop plants under alteration in environmental factors, especially those that are caused by climate change since these affect yields of crops. In this line, our work was intended to examine the composition and structure dynamics of the wheat phyllosphere microbiota under simulated soil flooding.

### Flooding severely affected leaf nutrient status

Flooding significantly reduced spring wheat performance as revealed by above- and belowground biomass changes, and it dramatically altered edaphic and root properties (see Francioli et al.^57^. Flooding had also a severe effect on leaf traits, as the concentration of all the measured nutrients were strongly affected by this stressor, especially at tillering and booting stage (Fig. S2). Flooded wheat plants showed a significant (P < 0.05) increase of leaf C, which could be due to the inhibition of photosynthate delivery from leaves to roots under flooding stress, thus resulting in carbohydrate accumulation in leaves of flooded plants^70,71^. Mn and Na concentrations were significantly (P < 0.05) higher in leaves of flooded plants at all the three PGS. These results support the view of an increased uptake of these elements and their accumulation in shoots under waterlogging^72^. On the contrary, leaf N, P, K, S and Mg concentrations were significantly (P < 0.05) reduced in flooded plants. Decreases of N and P in leaves during flooding have been already reported in previous studies as a consequence of a diminished uptake of these macronutrients from the flooded soil by plant roots^73–76^. Such a trend was also observed in our experiment (see Francioli et al.^57^). Furthermore, under oxygen deprivation plant roots may suffer of energy deficits which can compromise ion transport processes, such as the ability to translocate ions from roots to the leaves^77,78^, which might explain the lower concentration of Ca and Mg detected in leaves of flooded wheat plants. Moreover, root oxygen deficiency decreases the selectivity of K^+^/Na^+^ uptake by roots in favour of Na^+^ and retards the transport of K^+^ to shoots^79–81^, corroborating the significant increase of Na^+^ and the concomitant decrease in K^+^ observed in the leaves of wheat plants subjected to flooding. These findings highlight the profound effect of waterlogging on leaf nutrient status, and further supporting the view that flooding stress might induce a disruption of inherent root-shoot communication and promote a functional disequilibrium between roots and shoots^82^.

### Plant phenology drives leaf bacterial microbiota assembly

From all the leaf samples, we recovered 293,723 bacterial 16S rRNA gene high quality reads which clustered in 265 bacterial ASVs. Overall, bacterial sequences were affiliated to 7 phyla, 12 classes, 40 orders, 67 families and 92 genera. Proteobacteria was the most abundant phylum, comprising approximately 48% of the reads across all samples, followed by Actinobacteriota (30.4%), Firmicutes (10.1%) and Bacteroidota (6.9%) (Fig. S3). A small proportion of members of Patescibacteria, Gemmatimonadota and Deinococcota was also detected. In accordance with our results, phyla belonging to Proteobacteria, Actinobacteriota and Firmicutes were found to be the most abundant taxa in wheat leaf in greenhouse^83^ and field experiments^84^.

**Figure 1:**
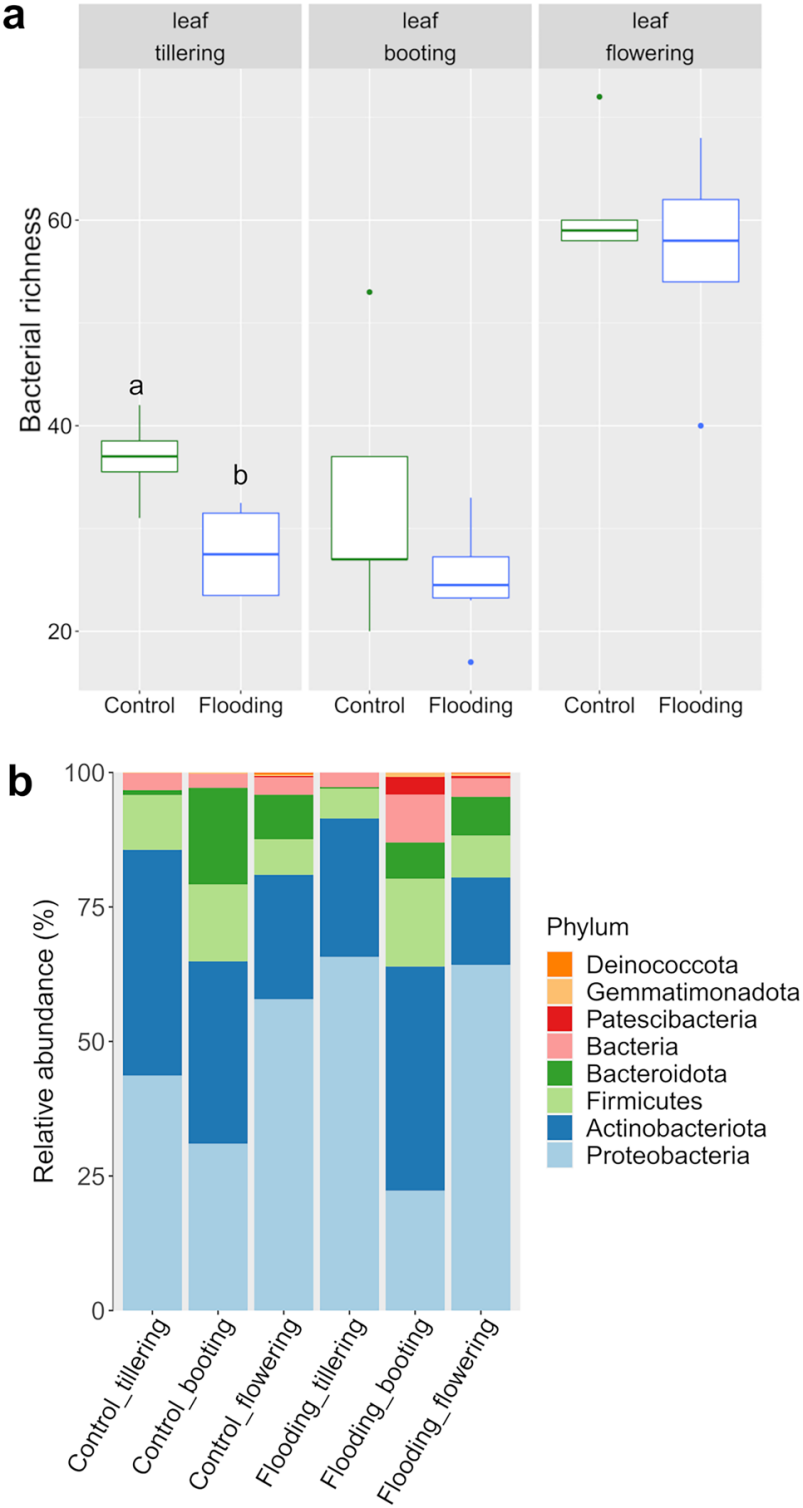
(**a**) Leaf bacterial richness and (**b**) relative abundances of the main bacterial phyla in the control and flooding treatment samples across plant grow stages (PGS). Different letters indicate significant differences in bacterial richness between water treatments within the same PGS based on Tukey’s HSD test P < 0.05.

Leaf-associated bacterial richness ranged from 17 to 76 ASVs and it differed significantly among PGS (P < 0.001, Table 1); i.e. flowering samples had on average the highest richness, regardless of treatment (Fig. 1a). Increases in bacterial richness and diversity over growing season have been observed in the phyllopshere of cereals^84^ and other crops^85^. This increase in bacterial richness might be linked with the ecological succession within the microbiota through plant development, as plant emerging surfaces create new habitat properties and thus, an expansion of niche breadth^86^. Therefore, along crop growth, the leaf microbiota diversity and complexity is expected to increase. Moreover, the observed increase in bacterial richness may be a direct response to signals between the plants and microorganisms, which could also mirror a specific responses to increasingly complex metabolites produced by mature wheat plants^87^. Interestingly, we found that flooding had a marginal effect on bacterial richness (Table 1), however, pairwise comparison within PGS revealed a significant decrease in bacterial ASVs in the flooded samples at tillering (Fig. 1a).

**Table 1:** The relative importance of plant growth stage (PGS) and watering treatment (WT) for the bacterial community richness associated with wheat leaves.

Parameter	df	Pseudo-F	R^2^	P‐value
PGS	2	52.098	0.778	0.001
WT	1	3.211	0.024	0.078
PGS * WT	2	0.291	0.004	0.759

**Figure 2:**
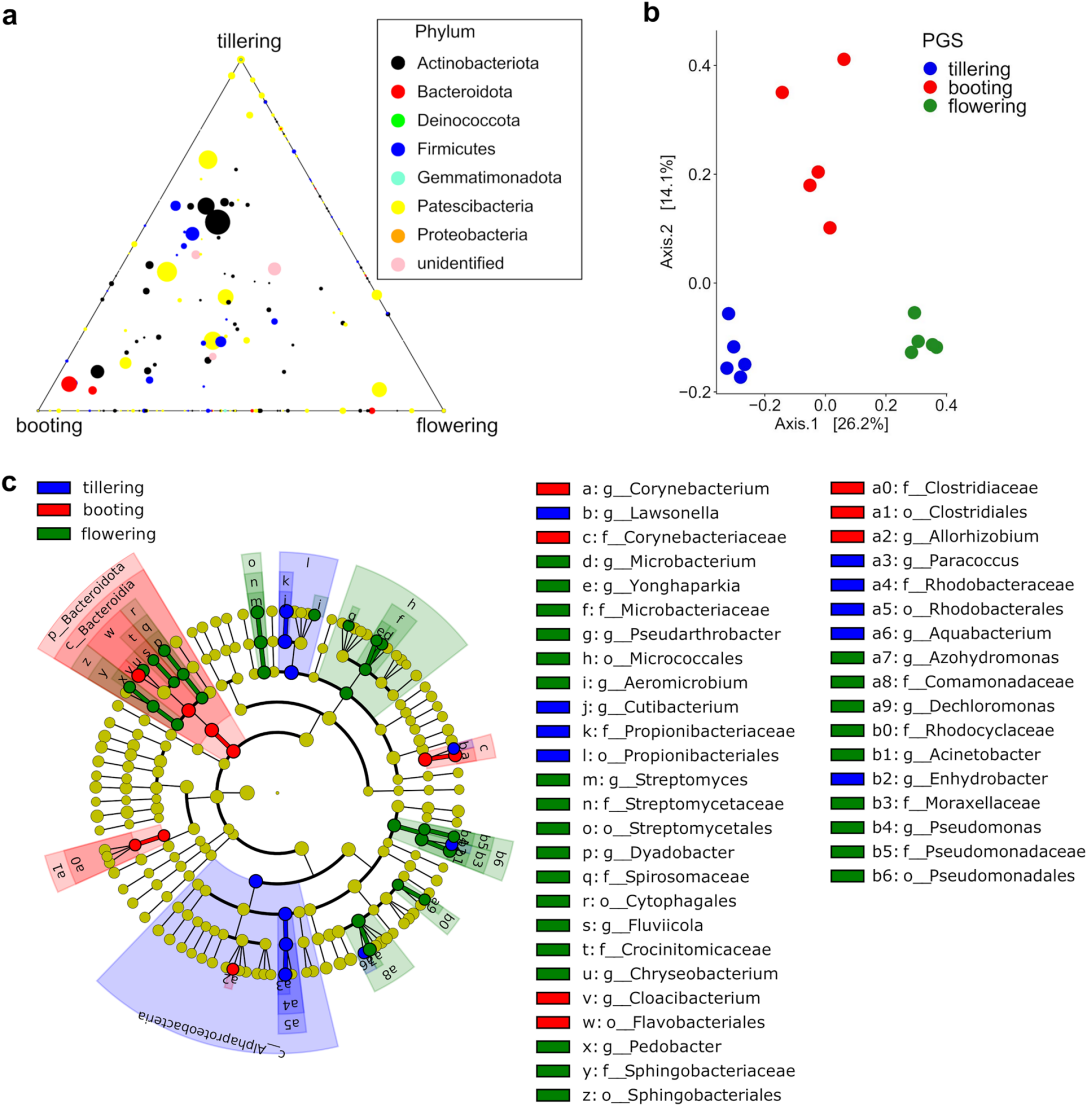
(**a**) Ternary plot of the bacterial ASVs distribution across plant growth stages in the leaf of wheat control plants; (**b**) Principal Coordinates Analysis (PCoA) using Bray–Curtis dissimilarity metrics of the leaf bacterial communities associated with the control treatment; (**c**) LEfSe analysis at multiple taxonomic levels comparing the bacterial community structure across PGS in control treatment. Cladogram illustrating the taxonomic groups explaining the most variation among the bacterial communities. Each ring represents a taxonomic level, with phylum (p), class (c), order (o), family (f) and genus (g) emanating from the center to the periphery. Each circle is a taxonomic unit found in the dataset, with circles or nodes shown in colours (other than yellow) indicating where a taxon was significantly more abundant.

Likewise, leaf bacterial beta-diversity was mainly affected by PGS, capturing 31.8% of community variation, while flooding had a direct marginal effect (Table 2). Plant phenology has been reported an important driver of leaf inhabiting microbiota^88,89^, and community shifts across plant growth stages are mainly attributed to changes in leaf physico-chemistry over time^90^. Thus, to better understand the effect of watering treatment in the phyllosphere microbiota, we sought to explore the temporal dynamics of the bacterial microbiota under controlled glasshouse conditions. Principal coordinate analysis on Bray–Curtis dissimilarities of the control samples clearly discriminated along the first coordinate the bacterial communities by PGS (Fig. 2b), highlighting the evident differences in community structure between the bacterial community structures at flowering from the other PGS (Figs. 1b, 2a,b). Indeed, higher dissimilarities in bacterial community structure between flowering and earlier PGS (booting, and tillering) were found (Figs. 2, 4), suggesting a different community assembly at the late growth stage flowering compared to mid- and early phase and confirming our first initial hypothesis. At the ASVs level, we found a high number of bacterial taxa uniquely detected in the leaf microbiota at flowering compared to the other two PGS (Fig. S4). These ASVs accounted for more than 25% of the total bacterial sequences in the control treatment, which further indicated a substantial restructuring of the bacterial microbiota between late and earlier PGS (Fig. S4). Dominant linear discriminant analysis (LDA) effect size (LEfSe) was employed to identify bacterial phylotypes as indicative biomarkers at each PGS, which are likely responsible for the large community shifts observed across PGS (Fig. 2c). For instance, bacterial members within the Alpha-proteobacteria phylum and the orders Propionibacteriales (Actinobacteriota) were significantly (P < 0.05) more abundant at tillering than in other PGS. The bacterial leaf microbiota at booting was characterized by a significant (P < 0.05) higher proportion of members affiliated with the Bacteriodota, but also with the Clostridiales (phylum Clostridia) and Corynebacteriales (Actinobacteriota). In contrast at flowering, the bacterial leaf microbiota was significantly (P < 0.05) enriched in taxa of Proteobacteria, in particular the orders Burkholderiales and Pseudomonadales. Moreover, significant (P < 0.05) enrichments in member of the Actinobacterial groups Streptomycetales (order) and Microbacteriaceae (family), and in the Bacteriodota orders Sphingobacteriales and Cytophagales characterized the leaf microbiota at flowering. At a finer taxonomic ranking, we observed a significant (P < 0.05) increase in the abundance of the bacterial endophytes affiliated with the Proteobacteria genera *Pseudomonas, Paracoccus*, *Aquabacterium*, *Enhydrobacter*, and with the Actinobacteriota genera *Cutibacterium* and *Lawsonella* in leaf samples at tillering. LEfSe identified the genera *Corynebacterium* (Actinobacteriota) and *Allorhizobium* (Proteobacteria) as biomarkers for the bacterial leaf microbiota at booting. Contrarily, the Actinobacteriota genera with putative endophytic capabilities *Streptomyces*, *Aeromicrobium*, and *Pseudarthrobacter*, together with the Proteobacteria genera *Dechloromonas* and *Acinetobacter* and the Bacteroidota genera *Pedobacter*, *Fluviicola* and *Dyadobacter* were significantly (P < 0.05) enriched at flowering. Leaf endophytes are an endosymbiotic group of microorganisms that colonize the leaf tissue, spending all or part of their life cycle within their hosts, and without causing any apparent symptoms of disease^91^. Bacterial endophytes have been reported to exert multiple effects on growth and health of several crop species, such as increasing N and chlorophyll content^92,93^, auxin indole-3-acetic acid (IAA) production^94,95^ and showed antimicrobial activity against several bacterial and fungal pathogens^96–98^. The significant differences in the leaf bacterial endophytic structure have usually resulted from leaf phenology^66,99^, and the results of the our study are in agreement with this observation, since it was found that the differences in the leaf endophytic microbiota were driven mainly by PGS. The evident shifts in abundance of PGS-specific bacterial genera across phenological stages might imply that specific leaf adaptation mechanisms occur at particular plant developing stage^33^, thus creating different temporal niches that are occupied by various taxa^100,101^.

**Table 2:** The relative importance of plant growth stage (PGS) and watering treatment (WT) for the bacterial community structure associated with wheat leaves.

Parameter	df	Pseudo-F	R^2^	P‐value
PGS	2	7.286	0.318	0.001
WT	1	1.796	0.039	0.046
PGS * WT	2	1.756	0.081	0.022

**Figure 3:**
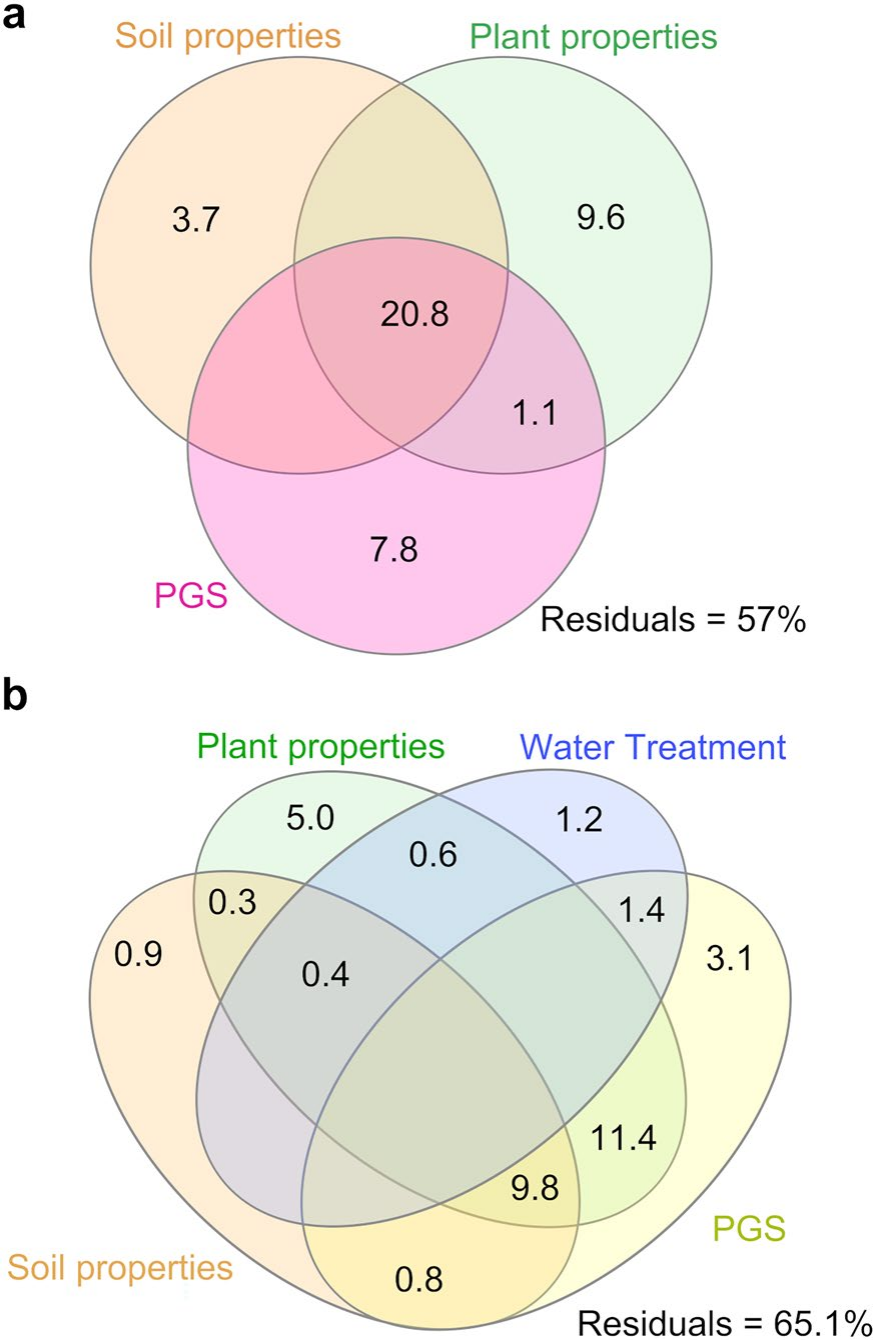
Variance partitioning illustrating the effects of the experimental parameters on the leaf bacterial community associated with (**a**) control and (**b**) both watering treatments (control and flooding samples).

The observed bacterial community shifts across PGS were found strongly associated with our experimental factors, as the bacterial leaf microbiota assemblage was significantly influenced by the pure effect of edaphic properties (4% of variance), plant traits (10% of variance) and PGS (8% of variance) and by their interactions (> 20% of variance) (Fig. 3a). This latter result indicated an important interactive effect of PGS with plant and soil attributes, which in turn significantly affected bacterial microbiota assembly in the phyllosphere. Indeed, shifts in soil and plant nutrient concentrations across the different PGS in the control plants were observed^57^ and db-RDA analysis revealed that root Na, leaf Na and total soil N were significant factors (P < 0.05) affecting leaf bacterial microbiota structure (Table 3). Plant traits, such as root and leaf nutrient concentrations, have been described as key factors in shaping the plant microbiota^102–104^, and their variations along plant phenology significantly influence bacterial community assembly^105,106^.

**Table 3:** Relationships between the predictor soil and plant properties with the leaf bacterial community in the control treatment only and in the full dataset (control and flooding samples).

	Control treatment		Full dataset	
	F	P	F	P
Leaf N	ns	ns	**3.216**	**0.001**
Leaf Na	**2.481**	**0.003**	**1.436**	**0.042**
Root Na	**3.480**	**0.001**	**4.435**	**0.001**
Root Mn	ns	ns	**1.526**	**0.021**
Soil N	**1.479**	**0.027**	ns	ns
Soil S	ns	ns	**1.977**	**0.003**

Results show marginal tests using the db-RDA model. Significant P-values less than 0.05 are indicated in bold. *ns* no significant.

### Flooding influenced significantly bacterial leaf microbiota assembly in young plants

Flooding had a detrimental impact on spring wheat fitness as reflected by a significant (P < 0.05) decrease of above and belowground plant biomass, especially at tillering and booting^57^, confirming the negative effect of flooding and soil waterlogging on wheat growth^107–110^.

**Figure 4:**
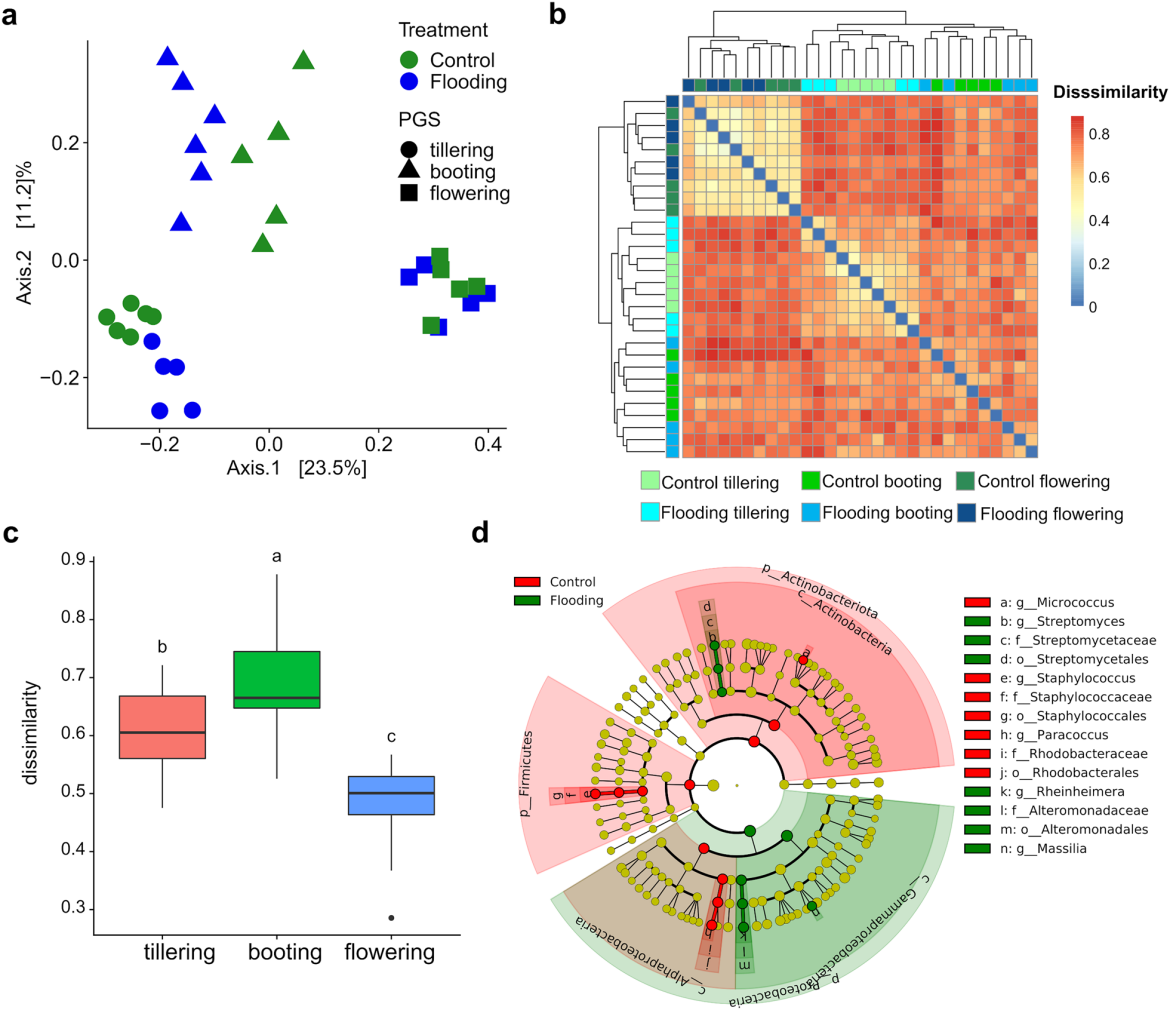
(**a**) Principal coordinates analysis (PCoA) on Bray–Curtis dissimilarity metrics of the leaf bacterial communities across PGS in the control and flooding treatment; (**b**) Heatmap representing the Bray–Curtis dissimilarity in bacterial community structure between watering treatment and PGS; (**c**) Box plots of Bray–Curtis dissimilarity index between watering treatment and PGS. The different letters indicate significant differences among plant growth stages (Tukey’s HSD test P < 0.05.); (**d**) LEfSe analysis at multiple taxonomic levels comparing the bacterial community structure between control and flooding treatment at tillering. Cladogram illustrating the taxonomic groups explaining the most variation among the bacterial communities. Each ring represents a taxonomic level, with phylum (p), class (c), order (o), family (f) and genus (g) emanating from the center to the periphery. Each circle is a taxonomic unit found in the dataset, with circles or nodes shown in colours (other than yellow) indicating where a taxon was significantly more abundant.

The leaf microbiota was also significantly affected by flooding and it explained 4% of variance (Table 2). We found a significant interaction between water treatment and PGS that accounted for an additional 8% of variation (Table 2). This latter result indicated a putative differential response to flooding along plant development. A principal coordinate analysis revealed a more pronounced effect of flooding on early than late PGS bacterial leaf microbiota (Fig. 4a). Analysis on structural dissimilarities of the bacterial microbiota between treatments at each PGS revealed a significant (P < 0.05) and larger impact of flooding stress at tillering followed by booting and flowering (Fig. 4b,c). These findings validated our second hypothesis, as they demonstrated that flooding caused a greater disruption to early bacterial leaf microbiota compared with late PGS. Recent studies have reported similar findings, i.e. that the juvenile plant-associated microbiota of the roots and rhizosphere were more affected by hydrological stress than those associated with later stages of plant development^57,111^. Taking together, all these observations may indicate that the phyllosphere microbiota of young plants is still in a dynamic process of establishment, in which community assembly is less resilient to abiotic and biotic stresses.

On the other hand, the microbiota associated with adult plants is relatively more stable due to prior establishment of a more stable community likely with a higher and tighter degree of interactions^7,112,113^. LEfSe further corroborated these observations, identifying in young plants (tillering) almost twice of bacterial biomarker taxa than at other PGS (Figs. 4d, S5). In particular, a depletion of Actinobacteriota, Firmicutes and Alphaproteobacteria together with an enrichment of Gammaproteobacteria were observed in the flooded treatments at tillering. At this PGS, flooding caused a significant increase (P < 0.05) in abundance of taxa associated with the genera *Streptomyces*, *Massilia* and *Rheinheimera* and a significant (P < 0.05) decrease in the proportion of *Staphylococcus*, *Paracoccus* and *Micrococcus*, which are bacterial endophytes that have been commonly isolated from wheat^114^ and other plants^115,116^. Taxa affiliated to *Massilia* are frequently associated with wheat^7,117^ and are considered putatively plant-beneficial due to their capability of producing proteases, sidephores and IAA^118,119^. We can speculate that their increase in leaf samples of flooding treatments at tillering might in our experiment had been leaf-mediated and buffered the plant physiological stress that has been induced by flooding.

At booting, flooding decreased significantly (P < 0.05) the abundance of *Streptomyces*, *Allorhizobium* and *Corynebacterium*, while an opposite trend was observed for *Aeromicrobium* and *Cutibacterium* (Fig. S5). Members affiliated to *Streptomyces* represent a well-studied genus of Actinobacteria with vast economic importance in agricultural plant production systems, because they include many potential biocontrol agents but also some important plant pathogens^120,121^. In recent studies, endophytic *Streptomyces* spp. were found to promote plant growth and protection against fungal disease by producing bioactive compounds in leaves of different wheat varieties^122,123^. As previously described, leaf developmental stage drives leaf microbiota assembly, and modulates metabolite accumulation and photosynthesis contributing to acclimation of plants to water stress^124^. Thus, the opposite response of *Streptomyces* to flooding observed at tillering and booting may suggest a specific responses of leaf inhabiting *Streptomyces* spp. to water treatment depending on PGS and leaf development.

At flowering, we observed the lowest differences in community structure between the flooded and the corresponding control treatments (Fig. 4c). This might further indicate that late PGS leaf bacteria microbiota is more stable and resilient to abiotic stresses compared to juvenile plants. Biomarker analysis revealed that flooding significantly (P < 0.05) decreased the abundance of Actinobacteriota and increased in the Alphaproteobacteria (Fig. S5).

Lastly, variance partitioning was employed to quantify the contribution of edaphic and plant properties, and their interactions with treatment and PGS on the structure of the bacterial leaf microbiota. These experimental factors captured together a large proportion of the variance, accounting for 35% (Fig. 3b). With the exception of PGS, which explained around 4% of variation, the pure effect of the experimental variables on the bacterial leaf microbiota structure was marginal, since most of the variance explained by them was shared. This indicated an interactive effect of PGS and water treatment on the plant and soil properties which in turn significantly affected the leaf microbiota assembly. Flooding dramatically altered various soil and plant traits. A significant increase (P < 0.05) in soil moisture, pH, Zn, and available P was observed^57^. Furthermore, we found a general and significant increase (P < 0.05) in leaf C, Mn and Na content under flooding, while leaf N, P, S, Mg and K showed an opposite pattern (Fig. S2). db-RDA analysis revealed that soil S, root Na and Mn concentration together with leaf N and Na concentration were significant (P < 0.05) factors affecting the bacterial microbiota structures in wheat leaf under flooding stress (Table 3). These findings are in line with recent works that highlighted the central role of soil and root traits in driving leaf bacterial microbiota assembly, together with leaf chemistry^27,69^. Overall, these observations acknowledged our second hypothesis, corroborating the fundamental influence of flooding on plant and soil properties, which in turn are firmly linked with leaf bacterial microbiota assembly.

## Conclusions

Leaf-inhabiting microorganisms play an important role in plant fitness, growth, and resilience to abiotic stress and resistance to pathogens. However, the identity of specific factors that govern these complex plant-microbiota interactions are largely unknown and their quantitative interactions in determining microbiota assembly in the phyllosphere are not well understood. Our study shed light on few of these aspects. This is the first work that resolved dynamics of the wheat’s bacterial leaf microbiota over plant development as affected by flooding. We proved that bacterial leaf microbiota assembly is mainly governed by plant phenology, with large differences in richness and structure between late and early plant and thus, leaf developmental stages. These microbiota structure shifts were primarily associated with changes in plant traits over time, such as the concentration of several macro- and micro-nutrients. Hence, our study underlines the importance of temporal sampling when studying plant-associated microbiota of annual plants, such as many crops. It seems essential to understand how plant microbiota are structured over the life cycle of their hosts, since the same stresses have different intensities and potential impacts at different PGS. Our study further explored the response of the leaf bacterial microbiota to flooding stress. The major outcome was that a more pronounced disruption in community assembly occurred in younger plants. At the late PGS, the leaf bacterial communities of both flooded and control treatments had a higher degree of similarity. Thus, those were likely more resilient to flooding stress.

Our study represents a fundamental starting point to understand the complex interaction between leaf microbes and crop hosts. Beyond, there is an urgent need to confirm and observe insights from controlled plant-level studies with field-level observations that include a broader variability of soil parameters and weather, but also further biotic interactions. Moreover, additional work is needed to fill the knowledge gaps on (i) how leaf metabolites changes when crop species are faced with extreme weather conditions, (ii) how these metabolites shape the phyllosphere microbiota diversity and composition, and (iii) the consequences for crop plant fitness, growth and eventually its yield.

## Supplementary Information

Supplementary Figures.

## Data Availability

All raw sequences were deposited in the European Nucleotide Archive (study accession number PRJEB50387). The data and the R-code used in the current study are available from the corresponding author on reasonable request.
