# Supplementary material for: Leaf bacterial microbiota response to flooding is controlled by plant phenology in wheat (Triticum aestivum L.)
Source: Sci Rep. 2022 Jul 1;12:11197. doi: 10.1038/s41598-022-15133-6 (PMC9249782; doi:10.1038/s41598-022-15133-6)
Supplement: Supplementary file 1 — Supplementary Figures. [file 41598_2022_15133_MOESM1_ESM.docx]

Supplementary Material

**Leaf bacterial microbiota response to flooding is controlled by plant phenology in wheat (*Triticum aestivum* L.)**

Running title: Bacterial community assembly in wheat phyllosphere

Davide Francioli^1^*, Geeisy Cid^2^, Mohammad-Reza Hajirezaei^2^ and Steffen Kolb^1,3^

^1^Microbial Biogeochemistry, Research Area Landscape Functioning, Leibniz Center for Agricultural Landscape Research e.V. (ZALF), Müncheberg, Germany

^2^Department of Physiology and Cell Biology, Leibniz Institute of Plant Genetics and Crop Plant Research, 06466 Gatersleben, Germany

^3^Thaer Institute, Faculty of Life Sciences, Humboldt University of Berlin, 10115 Berlin, Germany

* Corresponding author
*Davide Francioli
davide.francioli@zalf.de*

*Tel: +49 (0) 33432 82488*

**Figure S1. Experimental design of the greenhouse experiment conducted in this study.**


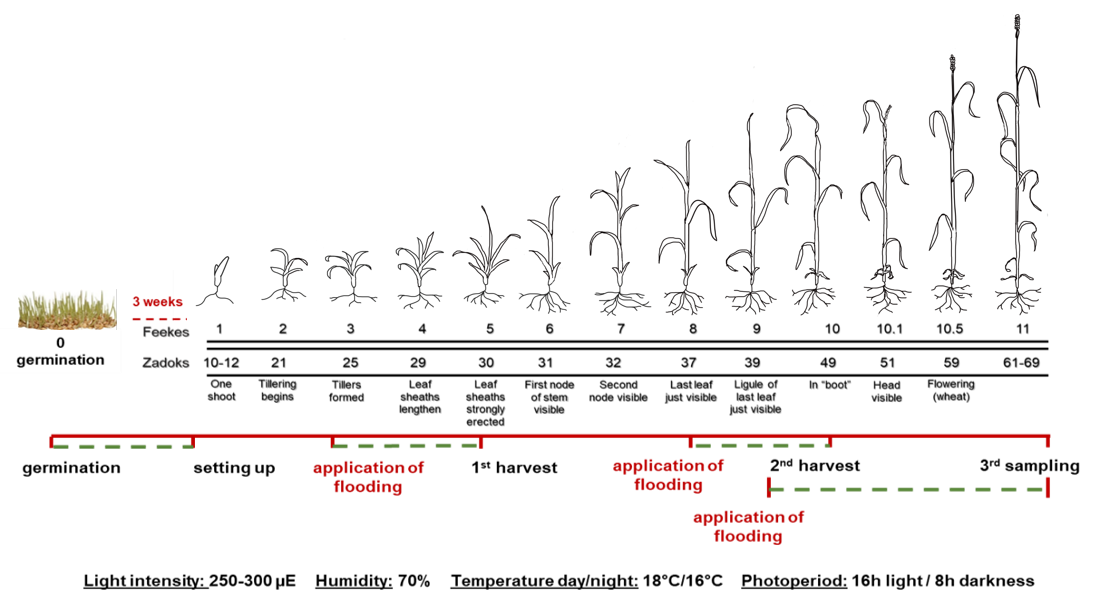


**Figure S2. Leaf traits measured in the different water treatments and PGS.** The different letters indicate significant differences within plant growth stages (Tukey’s HSD test *p* < 0.05.).


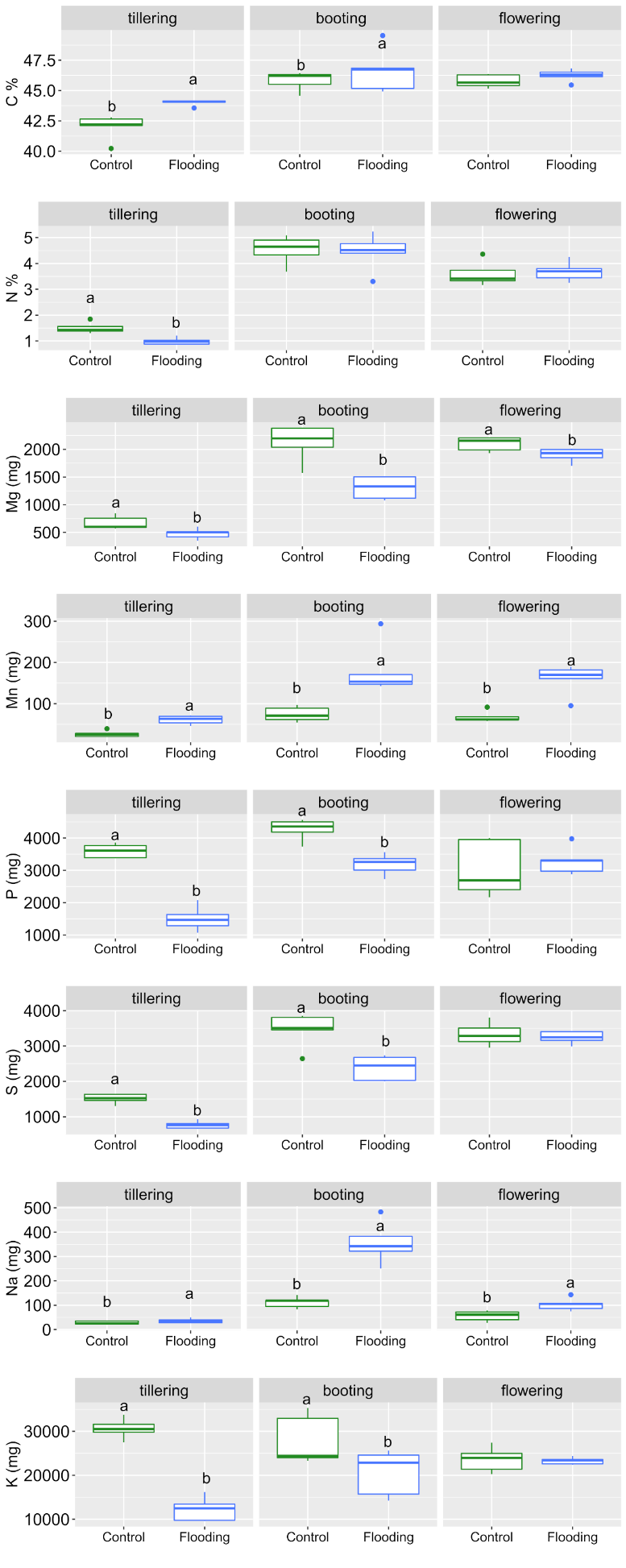


**Figure S3. Relative abundances of the bacterial major phyla in the leaf samples.**

**
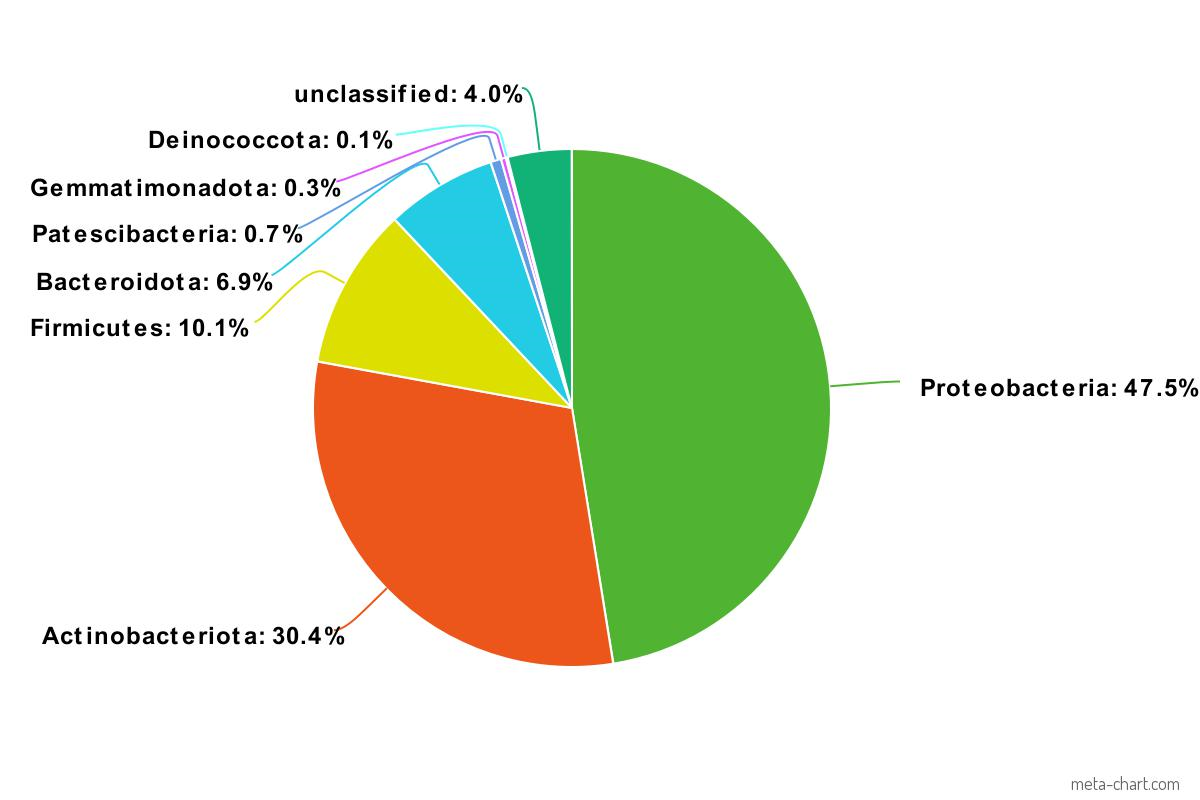
**

**Figure S4. Venn diagrams showing the unique and shared ASVs at each PGS of the leaf bacterial communities in the control treatment. Numbers represents ASVs, while in bracket is reported the cumulative relative abundance of the ASVs.**

**
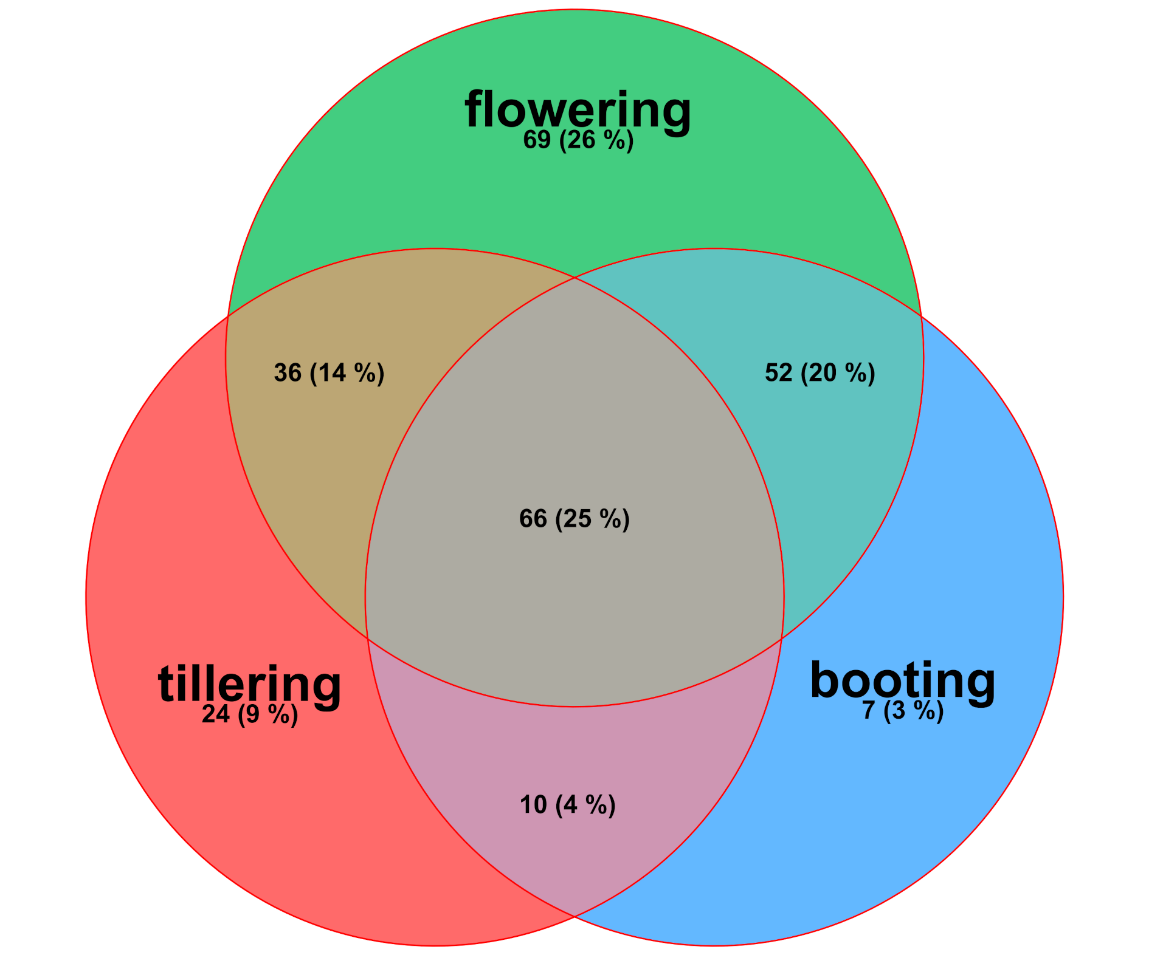
**

**Figure S5. LEfSe analysis at multiple taxonomic levels comparing leaf bacteria community structure in both control and flooding treatment at booting (a) and flowering (b). Cladogram illustrating the taxonomic groups explaining the most variation among leaf communities. Each ring represents a taxonomic level, with phylum (p), class (c), order (o), family (f) and genus (g) emanating from the center to the periphery. Each circle is a taxonomic unit found in the dataset, with circles or nodes shown in colors (other than yellow) indicating where a taxon was significantly more abundant.**

**
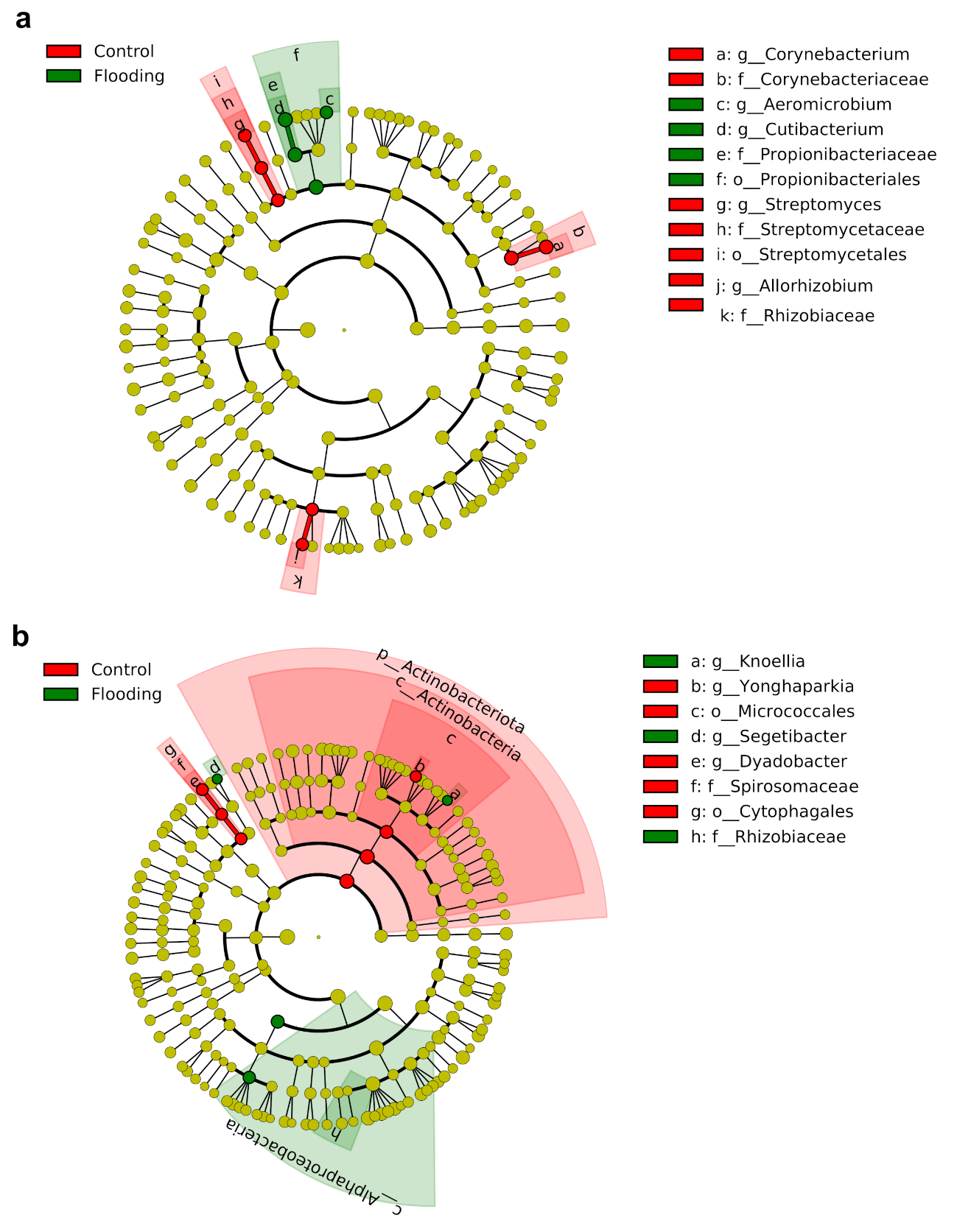
**
